# Reminder Cues Modulate the Renewal Effect in Human Predictive Learning

**DOI:** 10.3389/fpsyg.2016.01968

**Published:** 2016-12-20

**Authors:** Javier Bustamante, Metin Uengoer, Harald Lachnit

**Affiliations:** ^1^Department of Psychology, University of ChileSantiago, Chile; ^2^Faculty of Psychology, Philipps-Universität MarburgMarburg, Germany

**Keywords:** human learning, extinction, renewal, context, retrieval cue

## Abstract

Associative learning refers to our ability to learn about regularities in our environment. When a stimulus is repeatedly followed by a specific outcome, we learn to expect the outcome in the presence of the stimulus. We are also able to modify established expectations in the face of disconfirming information (the stimulus is no longer followed by the outcome). Both the change of environmental regularities and the related processes of adaptation are referred to as extinction. However, extinction does not erase the initially acquired expectations. For instance, following successful extinction, the initially learned expectations can recover when there is a context change – a phenomenon called the renewal effect, which is considered as a model for relapse after exposure therapy. Renewal was found to be modulated by reminder cues of acquisition and extinction. However, the mechanisms underlying the effectiveness of reminder cues are not well understood. The aim of the present study was to investigate the impact of reminder cues on renewal in the field of human predictive learning. Experiment I demonstrated that renewal in human predictive learning is modulated by cues related to acquisition or extinction. Initially, participants received pairings of a stimulus and an outcome in one context. These stimulus-outcome pairings were preceded by presentations of a reminder cue (acquisition cue). Then, participants received extinction in a different context in which presentations of the stimulus were no longer followed by the outcome. These extinction trials were preceded by a second reminder cue (extinction cue). During a final phase conducted in a third context, participants showed stronger expectations of the outcome in the presence of the stimulus when testing was accompanied by the acquisition cue compared to the extinction cue. Experiment II tested an explanation of the reminder cue effect in terms of simple cue-outcome associations. Therefore, acquisition and extinction cues were equated for their associative histories in Experiment II, which should abolish their impact on renewal if based on simple cue-outcome associations. In contrast to this prediction, Experiment II replicated the findings from Experiment I indicating that the effectiveness of reminder cues did not require direct reminder cue-outcome associations.

## Introduction

Background stimuli play a relevant role in the behavioral expression of learning. Extinction performance, for instance, seems to be particularly vulnerable to context changes (Bouton, 2004; Urcelay and Miller, 2014), as shown by the renewal effect. In a typical renewal procedure, a conditioned stimulus (CS; e.g., tone) is repeatedly paired with an unconditioned stimulus (US; e.g., shock) in Context A establishing conditioned responding (CR; e.g., fear) toward the CS. Then during extinction, the CS is presented repeatedly alone in Context B, which causes a gradual reduction in the response level elicited by the CS. Finally, when the participants are tested again in the acquisition Context A, the originally learned behavior reappears. This recovery effect is referred to as ABA renewal, with the letters denoting the contexts of acquisition, extinction, and test. Renewal has also been reported when acquisition, extinction, and testing take place in three different contexts (ABC renewal; Bouton and Bolles, 1979), and when acquisition and extinction are conducted in the same context and testing in a different one (AAB renewal; Bouton and Ricker, 1994). The renewal effect is a cardinal example for the persistence of expectations in the face of disconfirming information. The initially acquired expectations are not erased but suppressed instead by extinction. But this suppression is highly context-specific (Bouton, 1993, 2004).

The renewal effect is also considered as a model for relapse after exposure-based treatments (Bouton, 2000; Bouton et al., 2006). In exposure therapy, a patient is confronted with a problematic stimulus in order to decrease responding to it, for example, by exposing a phobic patient to the fear-eliciting event or stimulus. The renewal effect indicates that the therapeutic success in overcoming unwanted responses will be linked to a certain degree to the therapeutic environment. When a patient leaves the treatment context, relapse is facilitated.

Different strategies to influence the strength of renewal have been examined in the conditioning literature (for a review, see Laborda et al., 2011; Craske et al., 2014). One of these treatments is the use of reminder cues. For example, using a human fear conditioning task, Vansteenwegen et al. (2006) demonstrated that ABA renewal was affected by a reminder cue (a black cross) correlated with either acquisition or extinction. In one group, the reminder cue preceded the trials during the acquisition phase conducted in Context A, while in a second group the cue preceded the trials during extinction in Context B. Finally, all participants received presentations of the cue during a test of response recovery in Context A. Vansteenwegen et al. (2006) observed stronger renewal in those participants for which the cue was previously trained during initial acquisition than in those for which the cue previously accompanied extinction. Furthermore, the ability of reminder cues to modulate response recovery has been documented in a variety of preparations, including appetitive conditioning (Brooks and Bouton, 1994; Brooks, 2000; Brooks and Bowker, 2001) and ethanol tolerance (Brooks et al., 1999) in rats as well as fear conditioning (Dibbets et al., 2008; Dibbets and Maes, 2011), fear of spiders (Dibbets et al., 2013), and reactivity to alcohol-signaling cues (e.g., Collins and Brandon, 2002) in humans.

The aim of the present study was to extend the results of Vansteenwegen et al. (2006) to human predictive learning (Experiment I), and to examine a potential mechanism that may underlie the modulatory impact of reminder cues on response recovery (Experiment II). According to Brooks and Bouton (1993, 1994), there is the possibility that reminder cues might act through direct cue-outcome associations (e.g., Rescorla and Wagner, 1972). This view assumes that a cue presented in close temporal proximity to reinforcement of a CS acquires excitatory associative strength, while a reminder cue presented during extinction develops an inhibitory cue-outcome association. This view received support from a human fear conditioning experiment by Dibbets and Maes (2011) who observed that a cue presented during extinction of one CS attenuated conditioned responding to a second CS (summation test; Rescorla, 1969) indicating that the extinction reminder cue directly inhibited the US-representation.

Other studies, however, have shown that the effectiveness of reminder cues can be independent of any direct associations with the outcome. For example, it has been reported that an extinction reminder cue reduced response recovery even though it did not pass a summation test for conditioned inhibition (Brooks and Bouton, 1993; Dibbets et al., 2008). Furthermore, Brooks and Bowker (2001) showed that an extinction reminder cue still decreased response recovery after being paired with the US.

Experiment I was aimed at replicating the modulatory impact of acquisition and extinction reminder cues on response recovery reported by Vansteenwegen et al. (2006) for fear conditioning to human predictive learning, using a task with an ABC renewal procedure. Experiment II examined the importance of direct cue-outcome associations for the effectiveness of reminder cues. Therefore, we used an experimental design in which the acquisition and extinction reminder cues were equated for their associative histories. Each reminder cue was followed by the outcome on half of the trials, and was presented without the outcome on the other half. If the effectiveness of reminder cues relies on direct associations with the outcome, this treatment should abolish the impact of the cues on renewal. Both experiments were implemented in a predictive learning task that asked participants to imagine being a medical doctor whose patient often suffers from stomach trouble after the consumption of different meals in different restaurants (e.g., Üngör and Lachnit, 2006). The task was to predict the occurrence (+) or non-occurrence (-) of this stomach trouble. On successive trials, different stimuli (food types) were presented in one of several contexts (restaurants), and participants were asked to predict the patient’s reaction. On trials with a reminder cue, each food/restaurant presentation was preceded by a brief presentation of a picture showing either a cup of coffee or a glass of wine. During the learning phases of each experiment, each trial ended with information about whether stomach trouble had occurred or not.

## Experiment I

**Table 1** illustrates the design for the two groups of Experiment I. During Phase 1, all participants received Z+ trials in Context A (acquisition), with 80% of the trials preceded by a reminder cue (Y). During Phase 2, participants received training with Z- in Context B (extinction), with 80% of the trials preceded by a second reminder cue (X). Finally, during Phase 3 (Test) participants received trials with Z in Contexts B and C. For half of the participants (Group AC – acquisition cue) each of the test trials in Context C was preceded by the reminder Cue Y, the one presented during the acquisition phase, while for the other half of participants (Group EC – extinction cue) the trials in Context C were preceded by the reminder Cue X from the extinction phase. Thus, the Test consisted of an ABC renewal procedure, and each group was tested with a reminder cue correlated with either acquisition or extinction. If the reminder cues exert influence on responding during Test, we should find a lower level of renewal in Group EC than in Group AC.

**Table 1:** A summary of the experimental design of Experiment I (A, B, and C represent different restaurant names; Stimulus Z refers to the picture of a food item; Cues Y and X are pictures of two different drinks; + and - are occurrence and non-occurrence of stomach troubles, respectively; ?, participants received no feedback; the experimental design comprised additional filler cues that are not depicted in the table – see “Method” Section for details).

Group	Phase 1	Phase 2	Test
AC	A: Y/Z+	B: X/Z-	B: Z?
			C: Y/Z?
EC	A:Y/Z+	B: X/Z-	B: Z?
			C: X/Z?

### Method

#### Participants

The participants were 46 students from the Philipps-Universität Marburg, Germany (33 women and 13 men). Their age varied between 17 and 29 years, with a median of 22. They either were paid (€1.50), rewarded with chocolate or received course credits for participation. Participants were equally allocated to the different experimental groups as they arrived in the experimental room. They were tested individually and required between 10 and 15 min to complete the experiment. The data of 19 additional participants were excluded from the analyses because their predictions were incorrect on more than 30% of the trials with Stimulus Z during the last two blocks in Phase 1 and/or during the last two blocks in Phase 2. All participants gave their written consent to participate in the experiment.

#### Apparatus and Procedure

Instructions and all necessary information were presented on a computer screen. Participants interacted with the computer using the mouse. The following food types were used as stimuli: apples, avocados, bananas, broccoli, eggs, strawberries, carrots, corn, tomatoes, grapes, and lemons. The pictures of a glass of red wine and a cup of coffee were used as reminder cues. The names of three fictitious restaurants were used as contexts, labeled (translated from German) “To The Mug,” “By The Innkeeper,” and “In The Kettle,” written in red, blue, and green font, respectively. The assignment of the different food types to Stimulus Z and Filler Cues F1–F10 as well as the assignment of the restaurant names to the contexts were randomized for each participant. The pictures of the glass of wine and the cup of coffee were also randomly assigned to the acquisition and extinction cues. During the learning phases, each trial ended with either the presentation of the outcome (+; occurrence of stomach troubles) or with its absence (-; non-occurrence of stomach troubles).

Initially, each participant was asked to read the instructions (complete instructions attached as “Supplementary Material”). They were instructed to imagine being a medical doctor, and that one of their patients suffers frequently of stomach troubles after meals. Participants were told that their patient goes out often for meals to some restaurants. After each visit to a restaurant the participant would have to predict whether the patient suffers of stomach troubles or not.

Each trial started with a blank screen with a gray background presented for 500 ms followed by the name of one of the restaurants surrounded by a rectangular frame of the color associated with the restaurant. On trials with a reminder cue, in addition the picture of either a glass of wine or a cup of coffee was presented on the center of the screen. After 1000 ms, a picture of one food type replaced the reminder cue if it was present. The name of the food was written below the picture. Participants were told that their patient had eaten the food at the restaurant. They were instructed to make a prediction of whether they expect that their patient suffers from stomach troubles. Participants made their predictions by clicking on one of two answer buttons labeled “Yes, I expect stomach trouble,” and “No, I do not expect stomach trouble,” which were located below the food picture. Immediately after participants responded, another window appeared, telling the participants whether their patient suffered of stomach troubles or not. Participants had to confirm that they had read the feedback by clicking on an “OK” button. Then the next trial started.

During Phase 1 (see **Table 1**), all participants were given 10 trials of Z+ and F1- each in Context A, 10 trials of F2+, and F3- each in Context B, and 10 trials of F4+ and F5- each in Context C. The acquisition reminder Cue Y was present in 8 of the Z+ trials; the trials in which the reminder cue was shown were determined randomly. In Phase 2, all participants received 10 trials of F6+ and F7- each in Context A, 10 trials of Z- and F8- each in Context B, and 10 trials of F9+ and F10- each in Context C. The second reminder Cue X preceded Z- in 8 of the trials, assigned randomly. Trials with Stimulus Z in Phase 1 and Phase 2 that were not preceded by a reminder cue ensured that participants already experienced this stimulus in the absence of reminder cues prior to the Test (see below; see also, Brooks and Bouton, 1993, 1994; Vansteenwegen et al., 2006). Phase 2 followed Phase 1 without a break (the transition was not signaled to the participants).

Phase 1 and Phase 2 each were divided into five blocks, with each block consisting of two presentations of each food stimulus. The order of presentation of the trials within each block was determined randomly for each block and participant.

Phase 3 (Test) was introduced by instructions to the participants informing that the feedback would be omitted, but that they should try to predict the occurrence or non-occurrence of the outcome (complete instructions as “Supplementary Material”). Test trials were identical to learning trials, with the exception that the feedback window was omitted. All participants were presented with four Z trials in Context B and four trials with Z in Context C. For half of the participants (Group AC) each trial with Z in Context C was preceded by the acquisition Cue Y, whereas for the other half (Group EC) these trials were preceded by the extinction Cue X. The Test was divided into two blocks, and within each block each trial type was presented two times. The order of presentation of the trials within each block was determined randomly.

### Results

For this and the subsequent experiment, the 0.05 level of significance was employed for all statistical tests, and stated probability levels were based on the Greenhouse and Geisser (1959) adjustment of degrees of freedom where appropriate (for the sake of readability, we report uncorrected degrees of freedom). We report partial eta squared (η_P_^2^) as the measure of effect size.

#### Acquisition (Phase 1)

**FIGURE 1:**
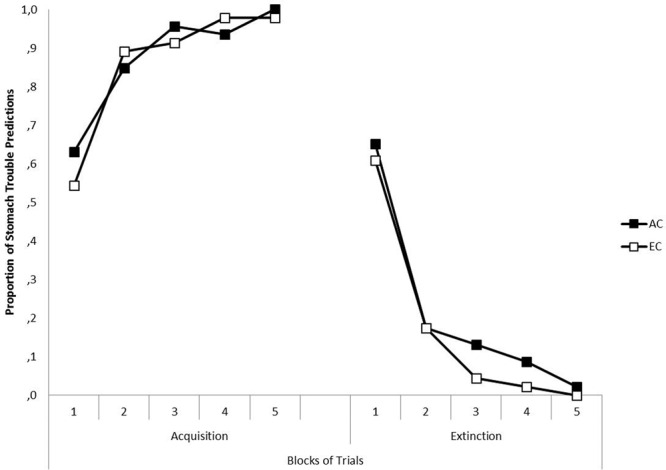
**The left-hand panel shows the mean proportion of predictions of stomach trouble in response to Z in Context A across five blocks in the acquisition phase of Experiment I, separately for Groups AC (black squares) and EC (white squares).** The right-hand panel shows the mean proportion of predictions of stomach trouble in response to Z in Context B across five blocks in the extinction phase of Experiment I for Groups AC and EC.

The left-hand panel of **Figure 1** presents for each group the mean percentages of stomach trouble predictions for Z+ in Context A across the five blocks of Phase 1. Black squares represent the data of Group AC, and white squares the data of Group EC. As can be seen, the mean prediction to Z+ increased across blocks, and there were no differences in responding to Z+ between groups. This was confirmed by a 5 × 2 (Block [1, 2, 3, 4, 5] × Group [AC, EC]) ANOVA. A significant main effect of Block was found, *F*(4,176) = 23.11, *p* < 0.001, η_P_^2^ = 0.344, indicating an increase of stomach trouble predictions to Z+ over the course of acquisition training, but neither a significant main effect of Group nor a significant Block × Group interaction was detected, all *Fs* < 1, showing that there was no difference in the predictions between groups.

#### Extinction (Phase 2)

The right-hand panel of **Figure 1** presents for each group the mean percentages of stomach trouble predictions for Z- in Context B across the five blocks of Phase 2. As depicted in **Figure 1**, the mean of stomach trouble predictions decreased across blocks, showing that the response to Z was successfully extinguished. This was confirmed by a 5 × 2 (Block [1, 2, 3, 4, 5] × Group [AC, EC]) ANOVA. There was a significant main effect of Block, *F*(4,176) = 54.40, *p* < 0.001, η_P_^2^ = 0.553, but no significant main effect of Group, *F*(1,44) = 1.78, *p* = 0.188, η_P_^2^ = 0.039, and no significant Block × Group interaction, *F* < 1, were detected, confirming that there were no differences between groups.

#### Test

**FIGURE 2:**
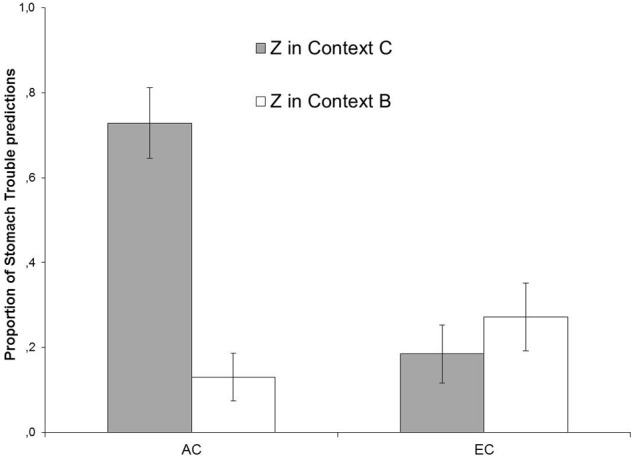
**Mean proportions of predictions of stomach trouble in response to Z during the test phase of Experiment I, collapsed across four presentations of each trial type separately for Groups AC and EC in Contexts B and C.** Error bars denote standard error of the means.

**Figure 2** depicts responding to Z in Contexts B and C during the Test in terms of the mean percentages of stomach trouble predictions, collapsed across the four test trials presented in each context. The left-hand bars present the predictions for Group AC and the right-hand bars show the predictions for Group EC.

As **Figure 2** demonstrates, participants in Group AC showed a higher level of responding to Z in Context C than in Context B (ABC renewal), while participants in Group EC showed similar levels of responding across the two contexts, indicating an absence of response recovery due to context changes. A 2 × 2 (Context [B, C] × Group [AC, EC]) ANOVA revealed a significant main effect of Context, *F*(1,44) = 12.38, *p* < 0.002, η_P_^2^ = 0.22, a significant main effect of Group, *F*(1,44) = 7.57, *p* < 0.009, η_P_^2^ = 0.147, and most importantly, a significant Context × Group interaction, *F*(1,44) = 22.24, *p* < 0.001, η_P_^2^ = 0.336, indicating that context-dependency of responding was stronger in Group AC than in Group EC. Further analyses were conducted on each group to explore the Context × Group interaction. A paired-samples *t*-test in Group AC yielded significantly stronger responding to Z in Context C than in Context B, *t*(22) = 6.45, *p* < 0.001, while there was no such a difference in Group EC, *t* < 1. These comparisons confirmed the presence and absence of renewal in Group AC and Group EC, respectively.

### Discussion

Taken together, after acquisition and extinction were conducted in two different contexts, testing the target stimulus in a third context disrupted extinction performance (ABC renewal) only if the test trials were preceded by a reminder cue related to initial acquisition training. When the test trials were preceded by a reminder cue related to extinction learning, however, extinction performance generalized perfectly to the third context.

The present results replicate the findings reported in human fear conditioning by Vansteenwegen et al. (2006) using an ABA procedure. The present results extend their findings to a human predictive learning procedure without biologically significant stimuli as well as to an ABC renewal design, both demonstrating the generality of the previous work.

In the learning phases of the present experiment, presentations of the acquisition reminder Cue Y were always followed by the outcome (occurrence of stomach trouble), while trials with the extinction reminder Cue X were consistently followed by its absence (non-occurrence of stomach trouble). When presented during Test, Y and X might have retrieved memories of their related outcomes which encouraged the participants to predict stomach trouble when the target stimulus was preceded by Y, and to predict its absence when the target was preceded by X. The purpose of the following experiment was to test this explanation in terms of direct reminder cue-outcome associations.

## Experiment Ii

**Table 2** depicts the design for the two groups of Experiment II. The learning and test phases were identical to those of Experiment I, with the exceptions that the acquisition reminder Cue Y additionally preceded 80% of the trials with F3- in Context B during Phase 1, and that the extinction reminder Cue X also preceded 80% of the trials with F6+ in Context A during Phase 2. Thus, in Experiment 2, acquisition and extinction reminder cues were equated for their learning histories in the way that each reminder cue was associated with the outcome on half of its presentations, while on the other half it was followed by the absence of the outcome. If reminder cues influence performance during the Test by retrieving memories related to their associated outcomes, then we should observe no difference in response recovery across the two groups in the present experiment.

**Table 2:** A summary of the experimental design of Experiment II (A, B, and C represent different restaurant names; Stimuli Z, F3, and F6 refer to pictures of different food items; Cues Y and X are pictures of two different drinks; + and - are occurrence and non-occurrence of stomach troubles, respectively; ?, participants received no feedback; the experimental design comprised additional filler cues that are not depicted in the table – see “Method” Section for details).

Group	Phase 1	Phase 2	Test
AC	A: Y/Z+	B: X/Z-	B: Z?
	B: Y/F3-	A: X/F6+	C: Y/Z?
EC	A:Y/Z+	B: X/Z-	B: Z?
	B: Y/F3-	A: X/F6+	C: X/Z?

### Method

#### Participants, Apparatus, and Procedure

The participants were 58 students from the Philipps-Universität Marburg, Germany (29 women and 29 men). Their age varied between 19 and 49 years, with a median of 22. The data of 21 additional participants were excluded from the analyses because their predictions were incorrect on more than 30% of the trials with Stimulus Z during the last two blocks in Phase 1 and/or during the last two blocks in Phase 2. All participants gave their written consent to participate in the experiment. The stimuli, instructions and procedure of Experiment II were the same as those of Experiment I, with the exceptions that the acquisition reminder Cue Y also preceded 8 of the 10 trials with F3- in Context B during Phase 1, and that the extinction reminder Cue X also preceded 8 of the 10 trials with F6+ in Context A during Phase 2. For each of the Stimuli F3 and F6, the trials in which the reminder cue was shown were determined randomly.

### Results

#### Acquisition (Phase 1)

**FIGURE 3:**
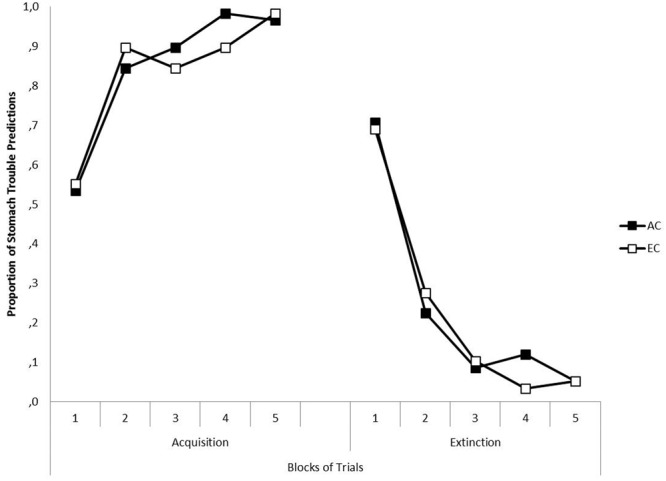
**The left-hand panel shows the mean proportion of predictions of stomach trouble in response to Z in Context A across five blocks in the acquisition phase of Experiment II, separately for Groups AC (black squares) and EC (white squares).** The right-hand panel shows the mean proportion of predictions of stomach trouble in response to Z in Context B across five blocks in the extinction phase of Experiment II for Groups AC and EC.

The left-hand panel of **Figure 3** presents for each group the mean percentages of stomach trouble predictions for Z+ in Context A across the five blocks of Phase 1. Black squares represent the data of Group AC, and white squares the data of Group EC. As can be seen, the mean prediction to Z+ increased across blocks, and there were no differences in responding to Z+ between groups. This was confirmed by a 5 × 2 (Block [1, 2, 3, 4, 5] × Group [AC, EC]) ANOVA. A significant main effect of Block was found, *F*(4,224) = 33.68, *p* < 0.001, η_P_^2^ = 0.376, indicating an increase of stomach trouble predictions to Z+ over the course of acquisition training, but neither a significant main effect of Group nor a significant Block × Group interaction was detected, both *Fs* < 1, showing that there was no difference in the prediction levels between groups.

#### Extinction (Phase 2)

The right-hand panel of **Figure 3** presents for each group the mean percentages of stomach trouble predictions for Z- in Context B across the five blocks of Phase 2. As depicted, the means of stomach trouble predictions decreased across blocks, showing that the response to Z was successfully extinguished. This was confirmed by a 5 × 2 (Block [1, 2, 3, 4, 5] × Group [AC, EC]) ANOVA. There was a significant main effect of Block, *F*(4,224) = 77.57, *p* < 0.001, η_P_^2^ = 0.581, but neither a significant main effect of Group nor a significant Block × Group interaction was detected, both *Fs* < 1, confirming that there were no differences between groups.

#### Test

**FIGURE 4:**
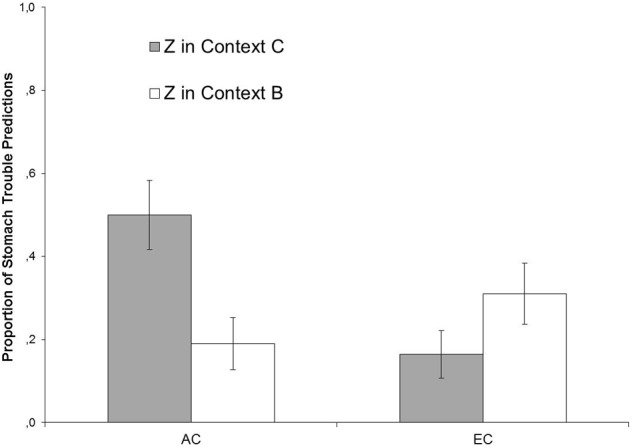
**Mean proportions of predictions of stomach trouble in response to Z during the test phase of Experiment II, collapsed across four presentations of each trial type separately for Groups AC and EC in Contexts B and C.** Error bars denote standard error of the means.

**Figure 4** depicts responding to Z in Contexts B and C during the Test in terms of the mean percentages of stomach trouble predictions, collapsed across the four test trials presented in each context. The left-hand bars present the predictions for Group AC, and the right-hand bars show the predictions for Group EC.

As **Figure 4** demonstrates, Group AC showed a higher level of responding to Z in Context C than in Context B, while Group EC showed similar levels of responding across the two contexts. A 2 × 2 (Context [B, C] × Group [AC, EC]) ANOVA revealed no significant main effect of Context, *F*(1,56) = 1.55, *p* = 0.218, η_P_^2^ = 0.027, no significant main effect of Group, *F*(1,56) = 2.11, *p* = 0.15, η_P_^2^ = 0.036, but there was a significant Context × Group interaction, *F*(1,56) = 12.09, *p* < 0.001, η_P_^2^ = 0.178, indicating that context-dependency of responding was stronger in Group AC than in Group EC. Paired-samples *t*-tests showed that participants in Group AC responded significantly stronger to Z in Context C than in Context B, *t*(28) = 3.35, *p* < 0.002, whereas there was no such difference in Group EC, *t*(28) = 1.57, *p* = 0.127.

### Discussion

The results from the Test of Experiment II were the same as those from Experiment I. Participants showed ABC renewal when testing occurred in the presence of a cue that had been experienced during initial acquisition learning. However, extinction performance was not disrupted by contextual changes when testing took place in the presence of a cue that had been administered during extinction treatment. In Experiment II, the two reminder cues did not differ with respect to their association with the outcome. Each reminder cue was paired with the outcome on half of its presentations. Thus, the modulation does not require direct reminder cue-outcome associations.

## General Discussion

In two human predictive learning experiments, we observed stronger response recovery following extinction when test trials were preceded by a reminder cue of initial acquisition compared to testing in the presence of an extinction reminder cue. Additionally, in Experiment II the acquisition and extinction cues were equated for their associative histories. Each reminder cue was followed by the outcome on half of the trials, indicating that the effect of the reminder cues does not require direct reminder cue-outcome associations.

Our study extends the generality of the conclusion drawn from previous experiments that the effect of a reminder cue can be independent of a direct association between the reminder cue and the outcome. Brooks and Bouton (1994) and Dibbets et al. (2008) found no evidence that an extinction reminder cue acquired inhibitory associative strength. Brooks and Bowker (2001) reported that an extinction cue did not lose its modulatory impact after being paired with the US. Our study is the first to provide evidence for this conclusion in a human predictive learning paradigm using an ABC renewal protocol. By equating the associative histories of the reminder cues, we extend the scope of methods demonstrating that the effectiveness of reminder cues is not necessarily a function of their own schedule of reinforcement.

Our results are rather consistent with the view that reminder cues modulate retrieval of entire CS–US associations akin to occasion setters (Holland, 1983, 1989; Rescorla, 1986; Schmajuk and Holland, 1998). An alternative explanation for the present results is provided by configural learning theories (Pearce, 1987, 1994). According to this view, the specific reminder cue-CS pattern might be encoded as a unique representation which would develop a direct connection to the US-representation. Future research might aim to differentiate between the configural and the occasion setting hypotheses, for example, by examining whether a reminder cue shows transfer of its modulatory properties to a second CS with an inconsistent reinforcement history, but not to other stimuli that were consistently paired with an outcome. This selective transfer is a hallmark of occasion setting (Holland, 1989) which cannot be explained by standard configural theories (Pearce, 1987, 1994).

The idea that reminder cues influence performance through their direct connections to the outcome cannot explain the results from our second experiment. However, this account provides a straightforward explanation of Experiment I. Therefore, we cannot exclude the possibility that reminder cue-outcome associations at least contributed to the recovery effects in the present study. In fact, there is some evidence for such a contribution when cross-experimental comparisons are taken into account. We observed stronger ABC renewal in Group AC from Experiment I than in Group AC from Experiment II. This was confirmed by a 2 × 2 (Context [B, C] × Group [AC/Experiment I, AC/Experiment II]) ANOVA revealing a Context × Group interaction, *F*(1,50) = 4.69, *p* = 0.035, η_P_^2^ = 0.086. This finding could be explained by assuming that the acquisition reminder cue in Experiment I acquired stronger excitatory strength than the one in Experiment II. However, we found no evidence for a contribution of direct cue-outcome associations in case of the extinction reminder cue. A 2 × 2 (Context [B, C] × Group [EC/Experiment I, EC/Experiment II]) ANOVA revealed no Context × Group interaction, *F* < 1. This latter finding is inconsistent with our analysis, but might also be considered to reflect a floor effect. Thus, the direct associations account could at least explain aspects of our data. However, conclusions from cross-experimental comparisons should be treated with caution, and future research will be required to investigate possible contributions of reminder cue-outcome associations to the strength of response recovery.

Our understanding of the mechanisms underlying the effectiveness of reminder cues has important implications for a clinical application (Craske et al., 2014). For instance, if an extinction reminder cue supports retrieval of the inhibitory CS–US association, this cue can be used as a powerful tool to enhance the long-term success of exposure-based treatments. However, if an extinction cue acts through a direct inhibitory connection to the US, then the cue should be removed from the clinical setting as it would be detrimental to the therapeutic goals. In this case, the cue would be a “safety signal,” for instance, signaling the absence of fear which would protect the fear-eliciting target stimulus from extinction.

In two experiments, we show that reminder cues exerted influence on the strength of response recovery following extinction in a predictive learning task. However, our experiments were not designed to assess the individual contributions of acquisition and extinction reminder cues to this behavioral modulation. The difference in response recovery during the test phase of each experiment might have been caused by (a) an increase of renewal due to the presentation of the acquisition cue, (b) a decrease in renewal by the extinction cue, or (c) both (see also Vansteenwegen et al., 2006). However, in each of our experiments, response recovery was completely abolished when testing was conducted in the presence of the extinction cue. Taken into account studies using a similar procedure demonstrating robust ABC renewal in the absence of reminder cues (e.g., Üngör and Lachnit, 2008), this diminution can be considered as indirect evidence that the extinction cue contributed to performance by reducing response recovery. However, future research is required to test this directly and to disentangle the individual and relative contributions of acquisition and extinction reminder cues on response recovery.

## Ethics Statement

The experimental procedure was approved by the ethics committee of the Psychology Department of the Philipps-Universitaet Marburg. Before the experiment, participants were informed about the general purpose and the general procedure of the experiment. And they were briefed that they can cancel their participation at any point during the experiment. Participants gave informed written consent to participate in the experiment. They confirmed their participation as voluntary and agreed to the use of their data in anonymous form for scientific purposes.

## Author Contributions

All authors listed, have made substantial, direct and intellectual contribution to the work, and approved it for publication.

## Conflict of Interest Statement

The authors declare that the research was conducted in the absence of any commercial or financial relationships that could be construed as a potential conflict of interest.
