# Supplementary material for: Reminder Cues Modulate the Renewal Effect in Human Predictive Learning
Source: Front Psychol. 2016 Dec 20;7:1968. doi: 10.3389/fpsyg.2016.01968 (PMC5167694; doi:10.3389/fpsyg.2016.01968)
Supplement: Supplementary file 1 [file Data_Sheet_1.DOCX]

Supplementary Material

Reminder cues modulate the renewal effect in human predictive learning

Javier Bustamante*, Metin Uengoer, and Harald Lachnit

*** Correspondence:** Javier Bustamante: je.bustamante@gmail.com

# General instructions for the experiment

## Initial Instructions

This study is concerned with the question of how people learn about relationships between different events. Imagine that you are a medical doctor and that one of your patients often suffers of stomach troubles after meals. Your task is to discover what causes this stomach troubles of which your patient is suffering.

Your patient likes to go out for meals. To The Mug, By The Innkeeper and In The Kettle are your patient’s favorite restaurants. You will be told which one your patient has visited each day and which food he has eaten there. Before some meals the patient also allows himself a drink (wine or coffee). Please look carefully at the drinks, foods and the respective restaurants. Thereafter you will be asked to predict whether the patient suffers of stomach troubles. For this prediction, please click on the appropriate prediction button. After you have made your prediction, you will be informed whether your patient actually suffered of stomach troubles. Use this feedback to find out what causes the stomach troubles your patient is suffering of. Obviously, at first you will have to guess because you don’t know anything about your patient. But eventually you will learn which causes lead to stomach troubles in this patient and you will be able to make correct predictions.

For all your answers accuracy instead of speed is essential. Please do not take any notes during the experiment. If you have any more questions, please ask now. If you don’t have any question, please start the experiment by clicking on the Next button.

## Instructions for the test phase

Now the feedback of whether your patient actually suffers from stomach trouble will be omitted. Nevertheless, please exert yourself to predict the occurrence or non-occurrence of stomach trouble as accurately as possible.

# Illustrations of the procedure

**
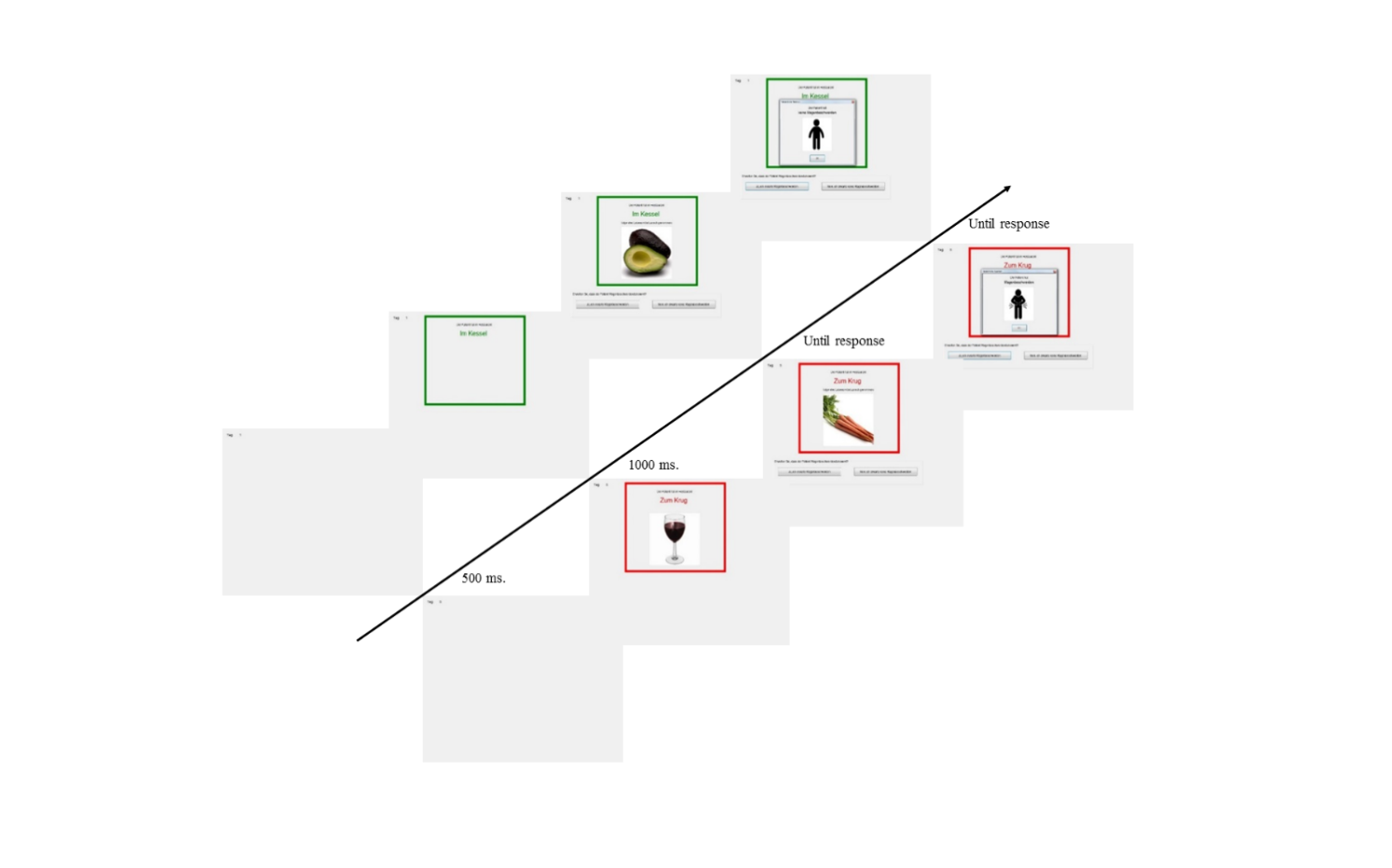
**

**Supplementary Figure.** The four pictures on the left show the parts of a trial without a reminder cue. The four pictures on the right show the parts of a trial with a reminder cue. Each trial started with a blank screen with a grey background presented for 500 ms. After this interval, the name of one of the restaurants appeared on top of the screen surrounded by a rectangular frame of the color associated with the restaurant. On trials with a reminder cue the picture of either a glass of wine or a cup of coffee was additionally presented on the center of the screen. Following an interval of 1000 ms, a picture of one food type was shown at the center of the screen, replacing the reminder cue if it was present. Below the picture the name of the food was written. Participants were told that their patient had eaten the food at the restaurant. They were instructed to make a prediction of whether they expect that their patient suffers from stomach troubles. Participants made their predictions by clicking on one of two answer buttons labelled “Yes, I expect stomach trouble”, and “No, I do not expect stomach trouble”, which were located below the food picture. Immediately after participants responded, another window appeared, telling the participants whether their patient suffered of stomach troubles or not. Participants had to confirm that they had read the feedback by clicking on an “OK” button. Then the next trial started.
